# Can Peak Expiratory Flow Measurements Differentiate Chronic Obstructive Pulmonary Disease from Congestive Heart Failure?

**DOI:** 10.1155/2012/912570

**Published:** 2011-10-27

**Authors:** John E. Gough, Kori L. Brewer

**Affiliations:** Department of Emergency Medicine, Brody School of Medicine, Greenville, NC 27834, USA

## Abstract

Dyspneic patients are commonly encountered by Emergency Medical Service (EMS). Frequent causes include Chronic Obstructive Pulmonary Disease (COPD) and Congestive Heart Failure (CHF). Measurement of peak expiratory flow rate (PEFR) has been proposed to help differentiate COPD from CHF. This prospective, cohort, pilot study was conducted to determine if PEFR in patients with an exacerbation of COPD were significantly different than CHF. Included were patients presenting with dyspnea plus a history of COPD and/or CHF. A PEFR was measured, values were compared to predicted average, and a percentage was calculated. Twenty-one patients were enrolled. Six had a diagnosis of COPD, 12 CHF; 3 had other diagnoses. Mean percentage of predicted PEFR with COPD was 26.36%, CHF 48.9% (*P* = 0.04). Patients presenting with acute COPD had significantly lower percentage of predicted PEFR than those with CHF. These results suggest that PEFR may be useful in differentiating COPD from CHF. This study should be expanded to the prehospital setting with a larger number of subjects.

## 1. Introduction

Dyspnea, a common cause of Emergency Medical Service (EMS) calls, can result from problems involving many organ systems. Some of the more frequently encountered scenarios are Chronic Obstructive Pulmonary Disease (COPD) and/or Congestive Heart Failure (CHF) which have different treatment regimens [1]. It is estimated that approximately 14 million Americans have COPD and five million carry the diagnosis of CHF [2]. These patients require rapid and accurate evaluation in order to provide the proper course of treatment. Studies have shown that physical examination alone is not always sufficient to determine the underlying cause of the patient's dyspnea. Both diagnoses may present with similar physical findings such as wheezing, rales, and jugular venous distension [3]. In the Emergency Department (ED), physicians have access to several diagnostic aids to assist in determining the cause of the dyspnea that are not available in the EMS setting. Peak expiratory flow meters are small, inexpensive, and accessible devices used to measure the peak expiratory flow rate (PEFR) after maximal inspiration. They are commonly prescribed for asthma patients but have shown promise in assessing COPD [4, 5]. Peak expiratory flow is not part of the standard protocol for gauging CHF patients, although several studies have suggested that PEFR may be useful in helping to differentiate cardiac from pulmonary causes of dyspnea [3, 5]. The purpose of this study is to determine if peak expiratory flow meters are effective in assisting EMS personnel in differentiating the respiratory and cardiovascular etiologies of dyspnea, and if so, to what extent. We hypothesize that the measured peak expiratory flow value calculated as a percentage of an established predicted value will be significantly lower in patients whose respiratory symptoms represent an exacerbation of COPD as opposed to CHF. As an initial step in testing this hypothesis, the authors chose to perform a data collection in the ED to determine if use of the PEFR may be useful in the prehospital setting.

## 2. Materials and Methods

This prospective, cohort, pilot study was conducted in the ED over a two-month period. A convenience sample of patients were enrolled based on their chief complaint and suspected history as recorded in triage or by EMS. Only those patients who presented with a history of COPD or CHF were included in the study. Patients were excluded if they were unable to cooperate with peak flow testing, or if any interventions, other than oxygen administration, were initiated by EMS, nursing or medical staff prior to obtaining the PEFR measurements. Once the patient was placed in a room, investigators obtained informed consent and a brief history. The patient was then given instructions on how to perform the peak expiratory flow test, and three attempts were made. The peak flow measurements were taken by one of the authors (J. E. Gough) or one of the study assistants trained in measuring PEFR. The treating physicians were blinded to the results of the PEFR testing. The highest peak expiratory value was recorded and considered the patient's best effort. This value was then compared to a standard predicted value based on the patient's height and age. A percentage of predicted value was calculated and used for the purpose of statistical analysis [6]. The patient's history, previous diagnoses, and discharge diagnosis were confirmed using medical records at a later date. The means of the percentages of predicted values were calculated and compared among the COPD and CHF subgroups. Statistical significance was determined using an unpaired *t*-test with *P* = 0.05 indicating a significant difference.

## 3. Results 

**Figure 1:**
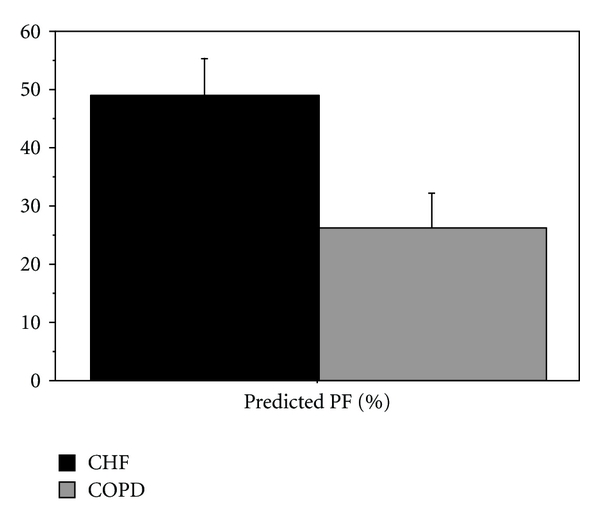
% Predicted peak flow measurement: COPD versus CHF.

Twenty-one total patients were enrolled in the study. The mean age for the patients enrolled was 61 ± 12.27 years. Six had a final diagnosis of COPD, 12 had a final diagnosis of CHF, and 3 had a different diagnosis. The mean % of predicted PEFR for patients with a history of COPD was 26.4 ± 14.3. For patients with CHF, it was 48.9 ± 22.6. The *P* value was calculated as 0.04 (Figure 1). Only those with diagnoses of CHF or COPD were included in the data analysis (Table 1). 

There were 6 patients enrolled which had a known history of COPD. These patients had a mean PEFR of 35.8%; all received a final diagnosis of COPD. There were twelve other patients with no history of COPD, and these patients had a mean PEFRF of 40.1%. There was no significant difference in peak flow between patients with a history of COPD and those without (*P* = 0.65).

Of the twelve patients with a known history of CHF, the PEFR of 48.8%, and 11 received a final diagnosis of CHF. The six patients enrolled who had no history of CHF had a mean PEFR of 26.3%. Those without a history CHF had a significantly lower PF than those with a history of CHF (*P* = 0.05).

One patient had a history of both CHF and COPD. The PEFR for this patient was 79.3%, and the diagnosis for this visit was CHF exacerbation. 

## 4. Discussion

Respiratory distress and shortness of breath are frequent complaints which account for approximately 13% of EMS transports [1]. Early appropriate treatment can significantly impact the patient's comfort as well as help prevent further deterioration and adverse outcomes. Studies have shown that both emergency physicians and EMS personnel can, in the majority of cases, make appropriate determination of the etiology of the patient's dyspnea on the physical exam findings. However, in approximately 30% of patients, the physical examination alone is not sufficient to identify the cause of the distress [3, 7]. Similar physical findings such as dyspnea, tachypnea, wheezing, cough, rales, and jugular venous distension may be seen in patients with both cardiac and pulmonary etiologies of dyspnea [3, 8]. Obtaining a past medical history from the patient and reviewing their medications may also yield clues. However, many patients carry multiple diagnoses. Studies have noted the prevalence of COPD to be as high as 20–32% in patients with known CHF [2]. This makes the distinction difficult, particularly in the prehospital environment. Making the appropriate assessment may be crucial, as incorrect treatment may lead to adverse outcomes for the patient. Diuretics used to treat cardiac dyspnea may cause electrolyte disturbances (mainly potassium and magnesium) and fluid loss [9]. Sympathomimetic amines and beta-agonists can cause tachycardia, increased QTC inducing arrhythmias, and hypokalemia. In the presence of underlying cardiovascular disease, these medications may increase the risk of heart failure and ischemic events including MIs [10].

Utilization of a peak flow test has been proposed as a simple, low cost, and time-effective instrument to help differentiate COPD from CHF as a cause of dyspnea [3, 4, 11]. In this paper the authors examined the mean percentage of predicted PEFR which was a different variable than in previous studies. While no absolute cutoff has been identified, these data support these previous studies' findings demonstrating that PEFR is significantly lower in patients undergoing a COPD exacerbation than that in those experiencing shortness of breath due to CHF. There was only one patient enrolled in this study with both COPD and CHF diagnoses previously documented. The PEFR in this patient was not significantly reduced (79.3%). This was consistent with the final diagnosis of CHF not a COPD exacerbation.

Limitations of this study include a small number of patients enrolled and the ED setting rather than EMS environment. The authors first sought to determine if the PEFR would potentially be useful before expanding the study to the prehospital setting. Study enrollment was limited by several factors, the first of which was the sampling method utilized as patients were only enrolled when one of the study investigators was present in the ED. Furthermore, a number of patients had interventions such as nebulizer treatments or medications initiated prior to obtaining PEFR. These limitations preclude the authors from determining how many patients were lost to enrollment. Although peak flow meters are easy to use and interpret, a major limitation to their use is that results are dependent on patients' understanding and effort [3, 5]. 

## 5. Conclusions

The results of this study demonstrate that in this population there is a difference between the PEFR values among patients with COPD as compared with CHF. Although the sample population is small, these data suggests that COPD patients show a lower percentage of predicted value for peak expiratory flow than those with CHF. This study should be continued in the prehospital setting with a larger sample size to better assess the utility of using this method among EMS personnel.

## Figures and Tables

**Table 1:** Patients enrolled in study.

Age	Predicted peak flow	Measured peak flow	% Predicted	Discharge Dx
64	550	132	24.0%	CHF
33	650	420	64.6%	CHF
87	402	175	43.5%	CHF
66	430	190	44.2%	CHF
41	650	370	56.9%	CHF
71	552	240	43.5%	CHF
71	416	70	16.8%	CHF
73	410	325	79.3%	CHF
57	605	180	29.8%	CHF
59	542	310	57.20%	CHF
59	542	310	57.20%	CHF
60	591	200	33.84%	CHF
65	550	125	22.7%	COPD
52	466	225	48.3%	COPD
64	572	85	14.86%	COPD
55	418	60	14.35%	COPD
50	602	240	39.87%	COPD
67	554	100	18.05%	COPD
